# Repositioning drugs for traumatic brain injury - N-acetyl cysteine and Phenserine

**DOI:** 10.1186/s12929-017-0377-1

**Published:** 2017-09-09

**Authors:** Barry J. Hoffer, Chaim G. Pick, Michael E. Hoffer, Robert E. Becker, Yung-Hsiao Chiang, Nigel H. Greig

**Affiliations:** 1 Department of Neurosurgery, Case Western Reserve University School of Medicine, Cleveland, OH USA; 2 Department of Anatomy and Anthropology, Sackler School of Medicine, Tel-Aviv University, Tel-Aviv, Israel; 3 Department of Otolaryngology, University of Miami Miller School of Medicine, Miami, FL USA; 4 Aristea Translational Medicine, Park City, UT USA; 5 Department of Neurosurgery, Taipei Medical University, Taipei, Taiwan; 6 Intramural Research Program, National Institute on Aging, National Institutes of Health, Baltimore, MD USA

**Keywords:** Traumatic brain injury, N-acetyl cysteine, Phenserine

## Abstract

Traumatic brain injury (TBI) is one of the most common causes of morbidity and mortality of both young adults of less than 45 years of age and the elderly, and contributes to about 30% of all injury deaths in the United States of America. Whereas there has been a significant improvement in our understanding of the mechanism that underpin the primary and secondary stages of damage associated with a TBI incident, to date however, this knowledge has not translated into the development of effective new pharmacological TBI treatment strategies. Prior experimental and clinical studies of drugs working via a single mechanism only may have failed to address the full range of pathologies that lead to the neuronal loss and cognitive impairment evident in TBI and other disorders. The present review focuses on two drugs with the potential to benefit multiple pathways considered important in TBI. Notably, both agents have already been developed into human studies for other conditions, and thus have the potential to be rapidly repositioned as TBI therapies. The first is N-acetyl cysteine (NAC) that is currently used in over the counter medications for its anti-inflammatory properties. The second is (−)-phenserine ((−)-Phen) that was originally developed as an experimental Alzheimer’s disease (AD) drug. We briefly review background information about TBI and subsequently review literature suggesting that NAC and (−)-Phen may be useful therapeutic approaches for TBI, for which there are no currently approved drugs.

## Background

### Traumatic brain injury

Traumatic brain injury (TBI) is the leading cause of death and long-term disability in the developed world. Annually, an estimated 10 million people suffer a TBI event worldwide [1, 2]. Projections indicate that TBI will comprise the third largest portion of the total global disease burden by 2020 [1]. Within the US, an estimated 1.7 million people per year sustain a TBI, and approximately 5.3 million people live with a TBI-induced disability [3, 4]. By far the majority of TBIs are mild to moderate in nature and account for 80–95% of cases, with severe TBI comprising the remainder [5]. With increases in survival rate following initial injury, TBI can result in substantial and lifelong cognitive, physical, and behavioral impairments that require long-term access to health care and disability services [5, 6]. Particularly vulnerable are the elderly, in which the same insult results in greater disability and can lead to a dramatic increase in the risk of neurodegenerative [7, 8] and neuropsychiatric disorders. TBI symptoms can occasionally resolve within the first year following injury, but some 70% to 90% of patients continue to exhibit prolonged and often permanent neurocognitive dysfunctions. It is now recognized that TBI is a time-dependent process, rather than a single static event. Emerging evidence indicates that this process can lead to early onset of dementia [7, 8]. From a clinical perspective, TBI is one of the most powerful environmental risk factors for development of Alzheimer’s disease (AD). Recent gene expression studies have defined the up-regulation of pathways leading to AD and Parkinson’s disease induced by mild, let alone moderate or severe forms of TBI [9–12]. In light of the lack of any available therapeutic options, it is important to understand the mechanisms that underlie head injury and the neuronal dysfunction and loss that ensue as well as possible therapeutics.

TBI-associated brain damage can be classified into two major phases. First, an initial primary damage phase occurs at the moment of insult. This includes contusion and laceration, diffuse axonal injury and intracranial hemorrhage, and results in instantaneous (necrotic) cell death [9, 13]. This period is followed by an extended second phase that encompasses cascades of biological processes initiated at the time of injury that may persist over much longer times consequent to ischemia, neuroinflammation, glutamate toxicity, astrocyte reactivity, axonal shearing and apoptosis [14–17]. Increasing evidence suggests that secondary brain injury may be reversible; depending on the biological cascades that drive the delayed secondary phase that occurs following TBI and how quickly and effectively these can be interrupted or mitigated [9, 18]. These cascades involve neuroinflammation, oxidative stress, generation of reactive oxygen species, inhibition of neurogenesis, apoptosis, loss of cholinergic circuits, and glutamate excitotoxicity. Importantly, these cascades occur in combination, rather than alone. Indeed, such combinations are likely complexed by time dependence, the nature of the TBI, the nature of the recipient and environmental factors. In the light of this, it perhaps not surprising that so many experimental therapeutics directed towards a single mechanism whose inhibition demonstrates promise in an animal model of TBI in a homogeneous rodent strain have failed to demonstrate efficacy in the human condition. In the section below, we summarize how NAC and (−)-Phen might alter these TBI-induced cascades and provide efficacy.

## N-acetyl cysteine

There is considerable literature on NAC as a neuroprotective agent in preclinical models of central and peripheral nervous system injury. NAC has been shown to have antioxidant and neurovascular-protective effects after preclinical TBI [19, 20]. NAC treatment following controlled cortical impact (CCI) increased levels of anti-inflammatory M2 microglia in white matter tracts [21]. Specifically, there is neuroprotective efficacy of a single dose of NAC in ameliorating biochemical and histological endpoints in a rat weight drop model [22] and of multiple doses in ameliorating inflammatory sequelae in an open skull dural impact rat model [19]. The antioxidant and anti-inflammatory effects of NAC [23–27] may be downstream consequences of inhibition of NAC-induced nuclear factor-κB-activated pathways that include cytokine cascades and phospholipid metabolism [28], which may also underlie the broader efficacy of NAC in rodent ischemia-reperfusion cerebral stroke models [24, 27, 29], a rodent sensory nerve axotomy model, and prevention of mitochondrial damage with loss of dendritic spines in hippocampal neurons [30]. Both NAC treatment alone and NAC treatment with topiramate ameliorate behavioral signs of mild weight drop TBI in rodent models [31].

**Fig. 1:**
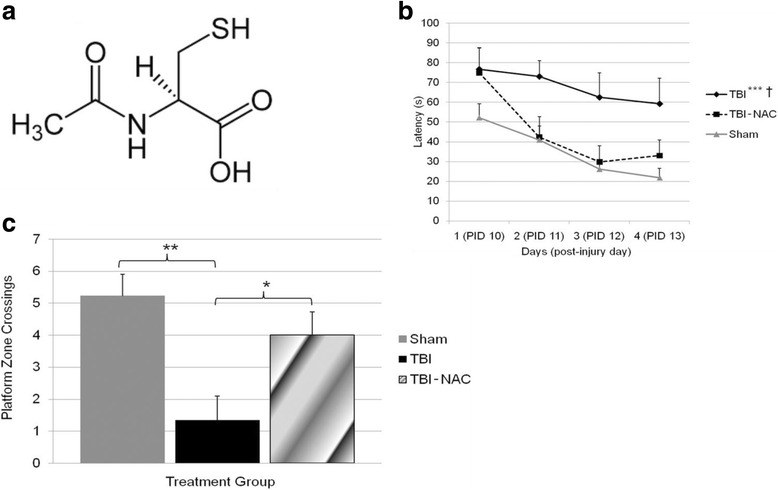
**a** Structure N-acetyl cysteine. **b** Post injury administration of NAC (50 mg/kg daily for 4 days) significantly improves MWM performance. MWM performance as measured by latency to reach the goal platform was compared between groups: TBI, TBI-NAC, and Sham. Both Sham and TBI-NAC groups have significantly shorter latencies to reach the goal platform as compared to the TBI group. Additionally, treatment with NAC after TBI improved performance in the MWM that reached sham levels. Data are presented as the mean ± SEM. *p < .05, ***p ≤ .001, sham relative to TBI. † *p* < .05 TBI-NAC relative to TBI. **c** Number of times animals crossed the within a 7.5 cm radius of the platform border during the probe trial. A one-way ANOVA showed significant differences between groups. Fisher’s LSD post hoc showed that sham and TBI-NAC (50 mg/kg daily for 4 days) had significantly better retention of the platform location as compared to TBI alone. Data are presented as the mean ± SEM. Brackets indicate comparisons between groups. *p < 0.05, **p < 0.01

The up-regulation of brain levels of glutathione (GSH) by systemic administration of NAC represents another potential neuroprotective mechanism. NAC is a precursor for GSH, which is a tripeptide derived by linking the amine group of cysteine to a glycine and to the carboxyl group of the glutamate side-chain. GSH is a critical intracellular antioxidant that prevents damage caused by reactive oxygen and nitrogen species (ROS and RNS). GSH is generated within its target cells from the amino acids, L-cysteine, L-glutamic acid and glycine. Importantly, the sulfhydryl (thiol) group (SH) of cysteine acts as a proton donor, and in this role is responsible for the antioxidant activity of GSH (Fig. 1). This cysteine represents the rate-limiting factor in cellular GSH production, since cysteine is relatively scarce, except in specific foods. Supporting the potential role of GSH in the effects of NAC, it has been reported that, in spite of its poor penetration into the central nervous system, NAC can significantly elevate GSH levels in the brain following oxidative stress [32, 33] and GSH deficiency [34]. Moreover, it has recently been demonstrated in a unique animal model of mTBI, involving thinning of the skull and compression, that GSH from the periphery can enter the brain and exert neuroprotective activity [35]. The cellular basis for memory and regulation of motivation associated with the nucleus accumbens may also be improved via NAC-induced neuronal activation of cysteine-glutamate exchange, augmented by the indirect effects of NAC on the metabolic glutamate receptors, mGluR2/3 and mGluR5, as reported for amelioration of cocaine-induced disruption of memory and regulation of motivation in rodents [36].

**Fig. 2:**
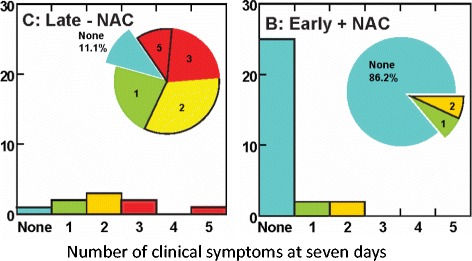
Number of clinical symptoms at seven days

We have also been evaluating NAC as a countermeasure for the neurosensory sequelae of mTBI in military personnel [37]. The rationale underpinning this approach is based on the fact that NAC’s mechanism of action can ameliorate or prevent the cascade of pathological events seen after mTBI, as noted above. In addition, NAC is the active ingredient in the brand name medication Mucomyst, a compound with a thirty-year safety history in U.S. hospitals, used for cystic fibrosis, acetaminophen poisoning, and high dye load x-rays as both an oral and an intravenous treatment. As such, our work represents repositioning of a “proven” medication whose tolerability/safety is well characterized, as opposed to introducing previously non-utilized or non-FDA approved pharmaceuticals. Historically, this has represented a more rapid and successful translational strategy than developing new previously untested drug candidates. Notably, we have demonstrated NAC to be efficacious in reducing the sequelae of mTBI in a randomized, double-blind, placebo-controlled study examining mTBI after blast injury [37]. Mild traumatic brain injury (mTBI) secondary to blast exposure is the most common battlefield injury in the Middle East. There has been little prospective work in the combat setting to test the efficacy of new countermeasures. The goal of our study was to compare the efficacy of NAC versus placebo on the symptoms associated with blast exposure mTBI in a combat setting. This study was a randomized double blind, placebo-controlled study that was conducted on active duty service members at a deployed field hospital in Iraq. All symptomatic U.S. service members who were exposed to significant blast and who met the criteria for mTBI were offered to participate in the study, and 81 individuals agreed. Individuals underwent a baseline evaluation and then were randomly assigned to receive either NAC or placebo for seven days. Each subject was re-evaluated at 3 and 7 days. Outcome measures were the presence of the following symptoms of mTBI: dizziness, hearing loss, headache, memory loss, sleep disturbances, and neurocognitive dysfunction. The resolution of these symptoms 7 days after the blast exposure was the main outcome measure in this study. Logistic regression on the outcome of ‘no day 7 symptoms’ indicated that NAC treatment was significantly better than placebo (OR = 3.6, *p* = 0.006). Secondary analysis revealed subjects receiving NAC within 24 h of blast had an 86% chance of symptom resolution with no reported side effects versus 42% for those seen early who received placebo. This study demonstrates that NAC, a safe pharmaceutical countermeasure, has beneficial effects on the severity and resolution of symptoms of blast induced mTBI. This was the first demonstration of an effective short-term countermeasure for mTBI (Fig. 2). Further work on long term outcomes and the potential use of NAC in civilian mTBI is warranted focusing on sports head injuries and traffic accidents. To highlight the value of work on NAC, the U.S. Army recently published its new strategic research plan for developing improved drug therapy for TBI [38]. In this document, the authors clearly indicate that NAC is one of the only safe medicines that has reasonable pilot data for the treatment of mTBI in a human, clinical setting and strongly recommend expanded clinical trials.

## (−)-Phenserine

Studies of experimental TBI models as well as post mortem human TBI samples have demonstrated losses in key features of the cholinergic system [39–42]. Cholinesterase inhibitors have, for example, been appraised in preclinical and clinical TBI studies, but have generated largely mixed results [43–46, 14, 47]. Paradoxically, rapid elevations in acetylcholine (ACh) levels within CSF of animal models and humans have been reported following TBI [48–51], with higher levels associated with greater injury [52]. This trend supported the early experimental and clinical use of anticholinergic agents, particularly muscarinic antagonists, for the mitigation of ACh-related toxicity to ameliorate TBI-induced deficits [53–57].

We evaluated the actions of an experimental and reversible anti-acetylcholinesterase (AChE) agent, (−)-phenserine tartrate ((−)-Phen) [58] in a well-characterized mild concussive model of TBI in mouse [59–64]. Notably, in addition to its anti-AChE activity, (−)-Phen is able to inhibit the synthesis of amyloid precursor protein (APP) and alpha-synuclein (α-syn), proteins of consequence in the pathology of AD and PD, respectively, and of currently increasing relevance to TBI in light of the up regulation of pathways leading to AD and PD in animal models of TBI [9–12] and in light of increased risk for early onset dementia and PD in humans suffering TBI [7, 8, 65–67]. In addition, (−)-Phen possesses anti-inflammatory properties [68], also a phenomenon of significance in TBI [69], although the majority of anti-inflammatory approaches have failed [70]. Furthermore, (−)-Phen possesses an array of trophic and anti-apoptotic actions via mechanisms that are now being characterized, as detailed below.

### The potential mechanisms for (−)-Phen to be repositioned for TBI are summarized as follows

**Fig. 3:**
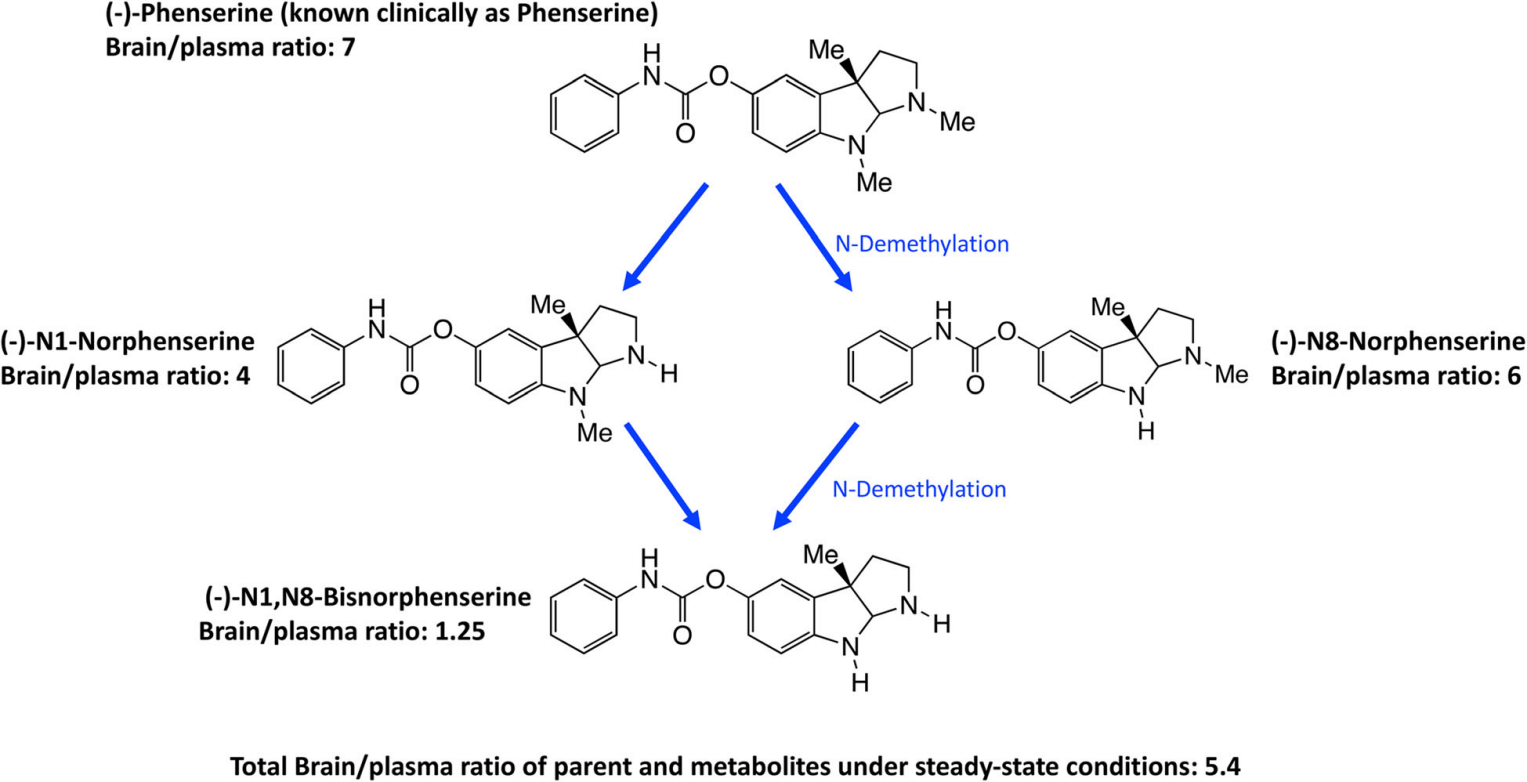
(−)-Phen is primarily metabolized by N-dethylation to yield (−)-N1- and (−)-N8-norphenserine that then are further N-dethylated to (−)-N1,N8-bisnorphenserine. Their brain/plasma ratios under steady-state conditions are shown

(−)- (−)-Phen, developed as a drug candidate for AD at the NIA, is a low molecular weight (mw 487.5), (−)- chirally pure, lipophilic (Log D 2.2) orally bio-available agent. The compound was originally developed as an acetyl- cholinesterase selective inhibitor with a high brain delivery [71–73]; importantly it is administered in the form of its tartrate salt to support its required aqueous solubility for pharmacological action [9]. In this regard, (−)-Phen and three active first-pass hepatic metabolites readily enter brain (approx. 7:1 to 1.25:1 brain/plasma ratios (Fig. 3) and, in dose-dependent relationships (EC_50_ = 26 to 100 nM), produce a broad range of pharmacological benefits of relevance to the effective treatment of disorders such as TBI and AD. The actions include anti-inflammatory; neutralizing oxidative stress; neuroprotection from anecrotic cell death and neuronal stem cell augmentation, as well as AChE, APP and α-syn inhibitions.

### (−)-Phenserine’s active metabolites

Preclinical and clinical studies have recently demonstrated that the broad range of beneficial pharmacological actions provided by (−)-Phen administration derive from the combined actions of (−)-Phen together with its stepwise metabolism to it primary metabolites (−)-N1- and/or (−)-N8-norphenserine to (−)-N1, N8-bisnorphenserine (also called (−)-N1,N8-bisnorphenylcarbamoyl-eseroline) [74, 75]. The differing plasma concentrations, brain:plasma distributions, t_1/2 elim_ rates, and ranges of EC_50_s of (−)-Phen and these key metabolites have been evaluated.

### (−)-Phenserine pharmacology

**Fig. 4:**
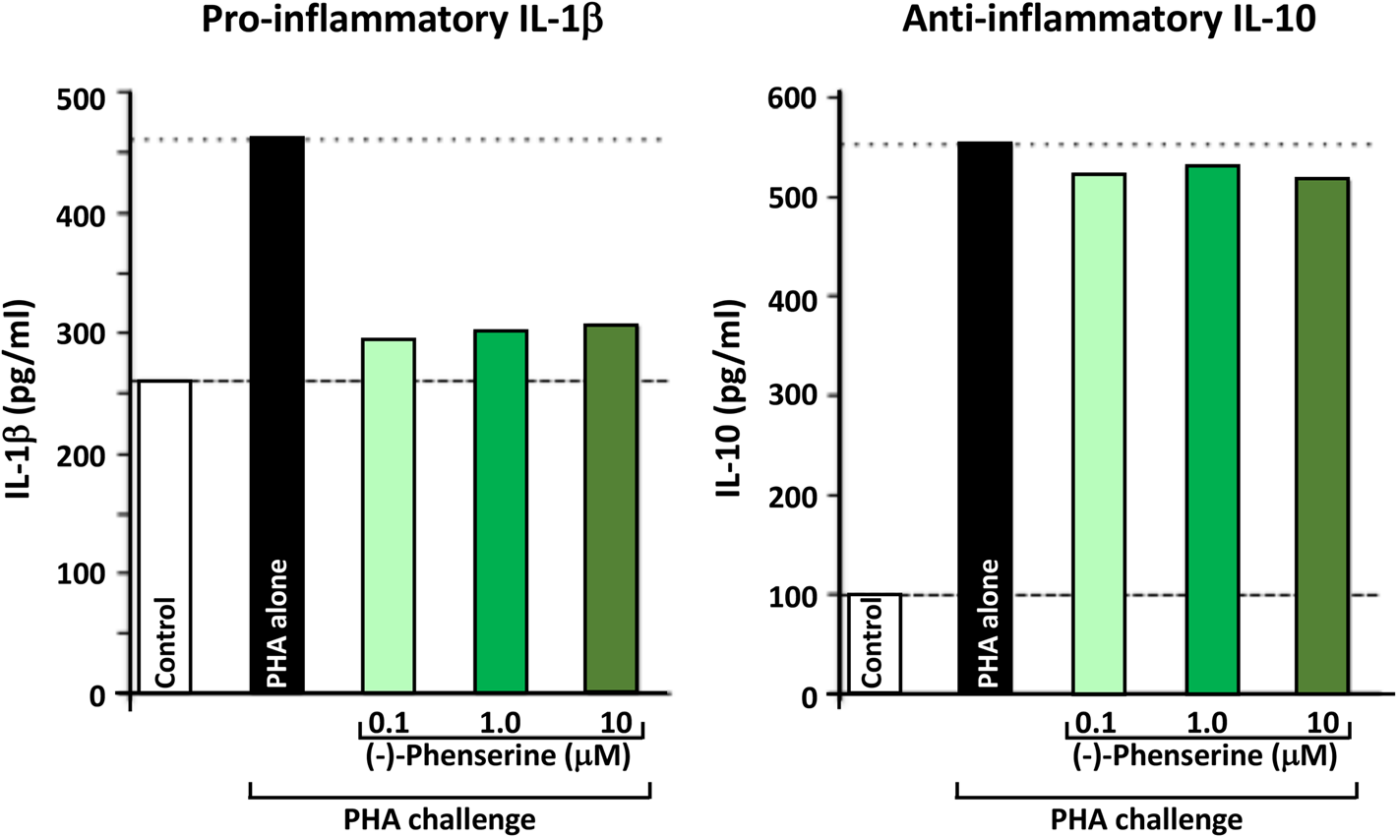
Human peripheral blood mononuclear cells (PBMCs) were isolated, from human blood, then cultured in the presence and absence of (−)-Phen for 24 h, and were then challenged with phytohemaggultanin (PHA: 3 μg/ml; Sigma-Aldrich) to induce inflammation and cytokine production. The detection limit for these assays is <1 pg/ml for IL-1β and <3 pg/ml for IL-10. The intra-and interassay CV was <10%. All results are expressed in pg/ml [68]

There is strong evidence of several relevant activities:

1. Antiinflammatory activities
  Phytohemagglutinin (PHA) is a lectin present in particular legumes, particularly red kidney bean (*Phaseolus vulgaris*), and has potent, cell agglutinating and mitogenic activities that cause the immune activation of peripheral blood mononuclear cells (PBMCs), and their subsequent generation of cytokines. PHA is often used as a tool to challenge PBMCs in culture, and as shown in Fig. 4, results in the production and secretion of pro- and anti-inflammatory cytokines, represented by IL-1β and IL-10, respectively. As illustrated in Fig. 4, (−)-Phen (0.1 to 10 uM) substantially mitigated the PHA-induced elevation in pro-inflammatory IL-1β levels without impacting PHA-induced anti-inflammatory IL-10 levels; thereby mitigating inflammation. Recent in vivo studies in experimental TBI demonstrate that the anti-inflammatory actions seen in ex vivo studies of PBMCs translate into animals by mitigating neuroinflammatory markers associated with microglial cell activation. In the light of extensive studies indicating that chronic neuroinflammation is a common characteristic across neurodegenerative disorders (including AD, PD, TBI and stroke) that drives disease progression, its mitigation by well tolerated agents can be considered beneficial [76].
2. Suppression of glutamate induced excitotoxicity:
  Glutamate is a key excitatory neurotransmitter in mammalian brain, and when intensely activated can be toxic to neurons over a range of acute CNS injury conditions that encompass TBI, stroke, hypoglycemia and status epilepticus. Excess glutamate is likewise implicated in chronic neurodegenerative disorders, particularly AD. Excessive glutamate activates its postsynaptic receptors, N-methyl-D-aspartate (NMDA), α-amino-3-hydroxy-5-methyl-4-isoxazolepropionate (AMPA) and kainate (KA). Such activation of AMPA receptors depolarizes the cell and concurrently unblocks the NMDA channels (releasing the Mg^2+^ block), and thereby allowing Ca^2+^ entry. Such depolarization opens voltage-activated calcium channels, causing Ca^2+^ ion and water influx into the cell down the osmotic gradient and leading the cells to cytotoxicity. Illustrated in Fig. 5, (−)-Phen treatment provides protection against glutamate-induced excitotoxicity in rat primary hippocamcal cultures. Specifically, glutamate significantly reduced cell viability of cultured primary hippocampal cultures by 53.5%, which was mitigated by (−)-Phen as a return to 73.5% of control levels and protection against anecrotic cell death [58]. Similar neuroprotection was found in human immortal neuronal (SH-SY5Y) cells with phenserine analogs following glutamate challenge.

**Fig. 5:**
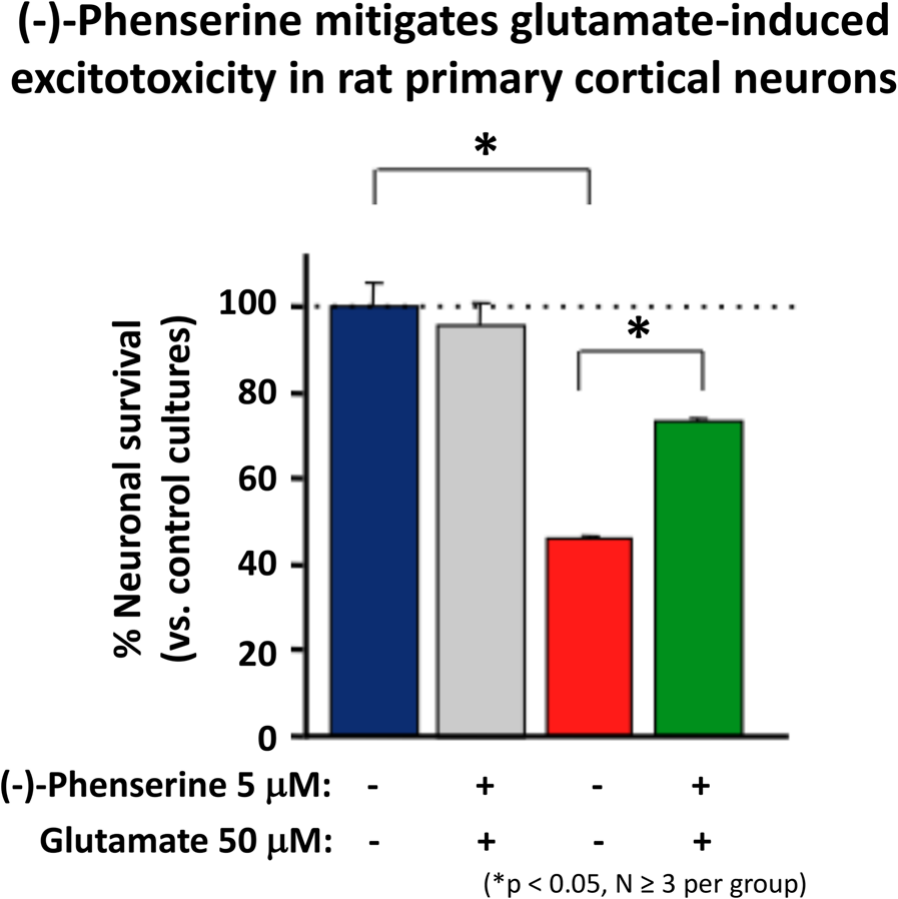
Cultured hippocampal neurons in triplicate were prepared from 18 to 20 day rat (Sprague Dawley) embryos and were cultured for 7 days. They were treated with (−)-Phen (5 uM) followed by addition of an excitotoxic concentration of glutamate (50 uM). Neuronal viability was assessed 24 h after addition of glutamate (MTS assay). The results are plotted as percent neuronal survival ± SEM [58]
    In relation to the in vivo relevance of cellular studies indicating protection against glutamate excitotoxicity, (−)-Phen has been evaluated in rats challenged with a lethal dose of the organophophate soman, where (−)-Phen both increased the survival rate of animals and provided neuroprotection of neuronal cells in the hippocampus, basolateral amygdala and cingulate cortex [77]. In soman-induced toxicity, the sudden substantial loss of AChE leads to abnormal accumulation of ACh within cholinergic synapses and results in the excessive stimulation of muscarinic and nicotinic receptors within the central and peripheral nervous systems. In the brain, such excessive stimulation of cholinergic neurons induces the release of glutamate, leading to the overactivation of the NMDA receptor, and excessive influx of Ca^2+^ resulting in excitotoxic neuronal cell death [77]. These studies, together, support the notion that neuroprotection provided by (−)-Phen in cellular studies is of in vivo relevance, as additionally supported in anoxia (stroke) in vivo studies in the rat.
3. Protection against oxidative stress:
  Fig. 6 shows that (−)-Phen provides protection against H_2_O_2_-induced oxidative toxicity in human immortal SH-SY5Y cells. Human SH-SY5Y cells were plated and after 24 h, cells were exposed to (−)-Phen (10 or 30 uM) followed by oxidative stress (100 uM H_2_O_2_). Cell viability was quantified at 24 h (MTS assay). (−)-Phen treatment significantly ameliorated the H_2_O_2_-mediated neuronal toxicity and provided protection against apoptotic cell death [58, 78].
4. Inhibition of APP synthesis:
  Multiple studies have demonstrated across different laboratories that (−)-Phen lowers levels of APP in neuronal cell cultures [79–81]. This appears to be a non-cholinergically facilitated action, as it is shared by its (+)-enantiomeric form, Posiphen ((+)-Phenserine tartrate) that lacks anticholinesterase activity, and is mediated post-transcriptionally via an iron response element within the 5′-untranslated region (5’UTR) of APP mRNA [79–81]. The EC_50_ of this APP lowering action appears to be in the order of 0.64 uM and 1.14 uM to lower secreted versus intracellular levels of APP, respectively, in human immortal neuronal (SH-SY5Y) cells [80]. Notably, primary neurons appear to be more sensitive, with (−)-Phen mediated APP lowering actions occurring at far lower drug doses (100 nM) [81]. As Fig. 7a and b document, (−)-Phen inhibits APP synthesis in vivo, and importantly lowers brain tissue levels of Aβ_42_. Fig. 7b shows the action of (−)-Phen on Aβ levels in the cortex of transgenic (APP_SWE_ + PS1) AD mice over-expressing human Aβ, in which a daily dose of 2.5 mg/kg substantially (*p* < 0.05) lowered APP as well as Aβ. Such (-)-Phen induced APP lowering action in brain translated to rats (Fig. 7a). By contrast, neither donepezil nor (−)-physotigmine (a structural analog of (−)-Phen) shared this action. In line with the described APP lowering actions of (−)-Phen, a similar dose in rats lowered nucleus basalis lesion-induced elevations in APP, as evaluated in CSF samples [82]. Figure 8 shows the suppression of Aβ_42_ after administration of (−)-Phen to humans in a study of healthy volunteers administered the agent twice daily over 35 days in which the dose was elevated to 15 mg BID ([83], Fig. 8). This same (−)-Phen dose provided an efficacy signal in mild to moderate AD patients [73]. A proof of mechanism clinical study of Posiphen likewise has demonstrated APP and Aβ lowering actions, as evaluated in time-dependent CSF samples obtained after 10 day dosing. Notably, (−)-Phen’s APP lowing action appears to be shared by not only its [+]-enantiomer but also by its 3 primary metabolites at concentrations as low as 100 nM [81]. Furthermore, these actions on APP by (−)-Phen and analogs additionally result in significant reductions in α-syn, which similarly appears to have a regulatory element controlling its translational efficiency within its 5’UTR [84–86].

**Fig. 6:**
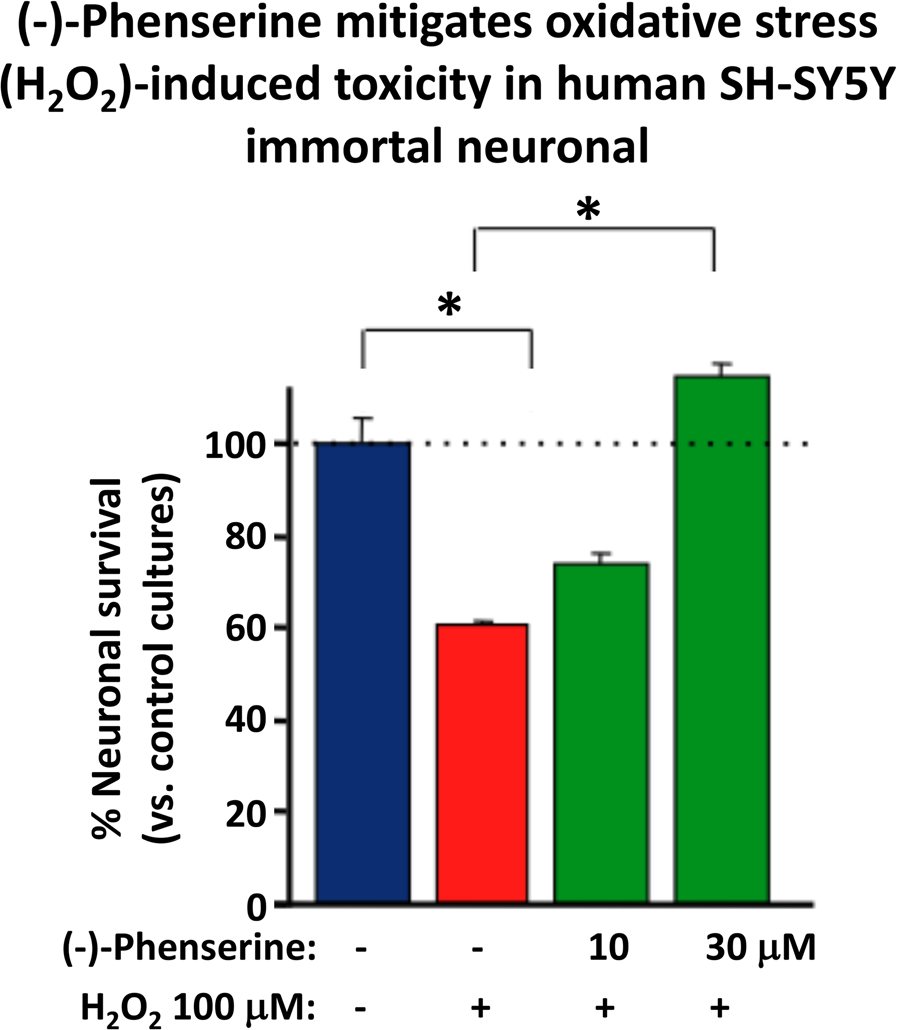
Human SH-SY5Y were treated with and without (−)-Phen and challenged with oxidative stress (H2O2: 100 uM). Cell viability was quantified by MTS assay at 24 h. * designates comparisons with cells challenged with H2O2 (*p < 0.05, *N* ≥ 4 per group). Viability with (−)-Phen (30 uM) treatment was no different from control unchallenged cells

**Fig. 7:**
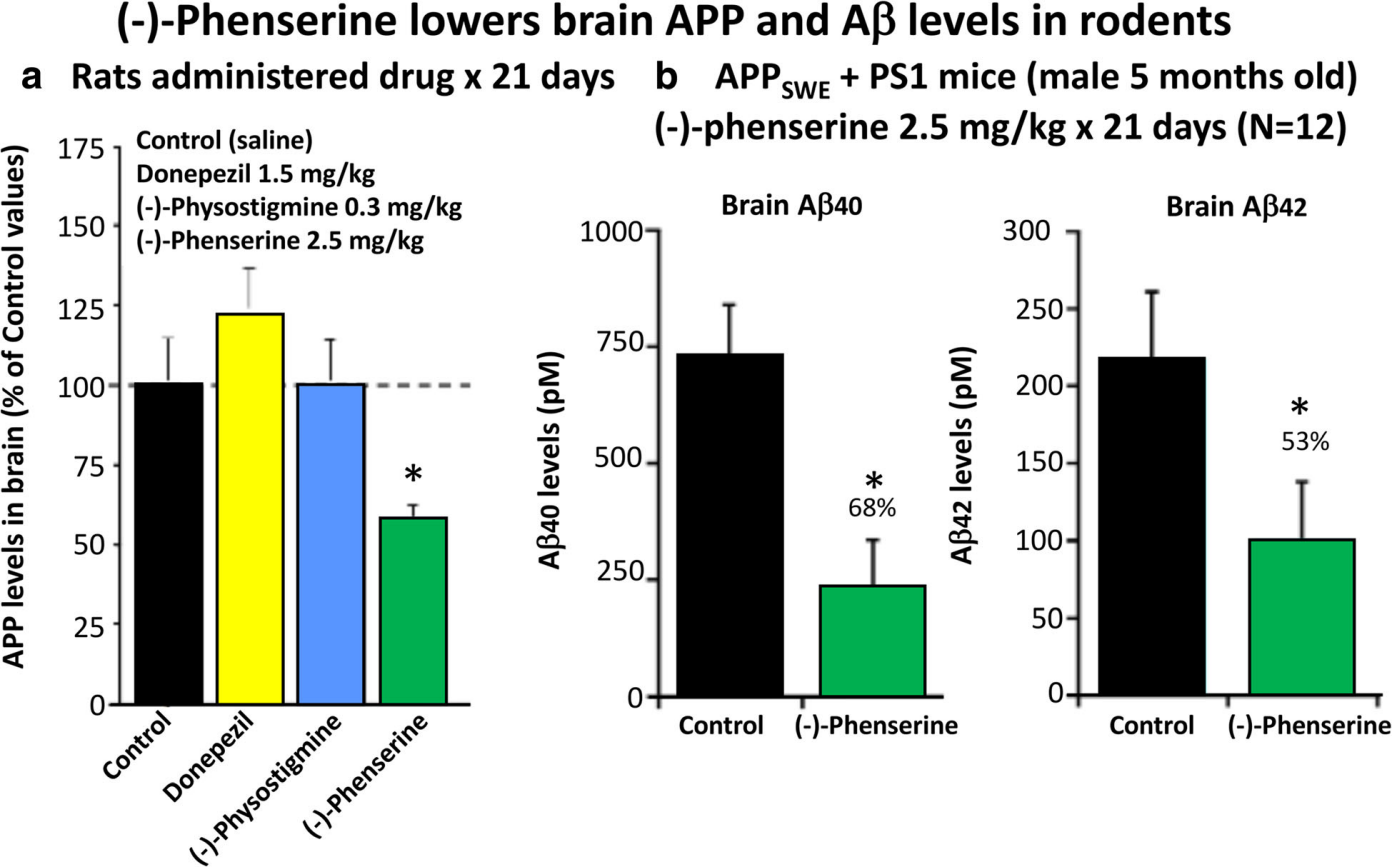
**a** and **b** (−)-Phen (2.5 mg/kg, i.p.) was administered for 21 consecutive days to rats and mice. Animals were killed within 2 h of their final (−)-Phen dose or saline; a brain (cortex) sample was taken and immediately frozen to –70oC and thereafter analysed for Aβ by ELISA. (−)-Phen significantly (*p* < 0.05) lowered APP, Aβ (1-40 and 1-42) levels vs. controls. This decline, particularly in Aβ42 levels, was likewise found in wild type mice dosed with (−)-Phen (2.5 mg/kg and 7.5 mg/kg, i.p.) for 21 days [80]

**Fig. 8:**
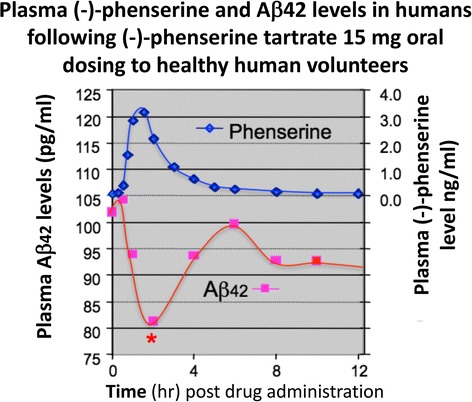
Administration of (−)-Phen to humans, by gradually escalating the dose to achieve 15 mg BID resulted in a decline (~20%) in levels of Aβ42 evaluated in the plasma time-dependently following the final (−)-Phen dose. This reduction coincided with the achieval of peak plasma (−)-Phen concentrations and suggests that the maintenance of long-term steady-state levels of (−)-Phen by slow-release formulations could provide a sustained lowering of Aβ42 in humans [83]. Phenserine tartrate was administered orally to healthy volunteers. Days 1-28: 10 mg BID; Days 29-34: 15 BID; Day 35: 15 mg. Blood samples were drawn on Days 1, 28, 29 and 35. Plasma samples were analyzed for Aβ1-42 using a sandwich ELISA. Plasma phenserine concentrations were determined by LC/MS/MS
    In the light of several epidemiological studies reporting that a history of brain trauma places a patient at greater risk of developing AD and/or PD [7, 8, 65, 66, 67], (−)-Phen mediated reductions in APP, Aβ and α-syn may translate into potential therapeutic value. In this regard, diffuse axonal injury (DAI) is one of the most frequent and key pathologies that occurs in TBI in both humans and animal models (87). APP, in particular, is routinely present in high concentrations in axons and is conveyed through neurons via fast axonal transport. As a consequence, a rapid and substantial accumulation of APP is routinely evident in damaged axons following experimental and human TBI. In fact, immunohistochemical evaluation of APP accumulation associated with axons, particularly in white matter regions, is routinely used to detect DAI in human brain tissue [87]. The accumulation of APP in axons following TBI is considered an early event, and is associated with upregulated APP gene expression [88, 89, 90]. The extensive co-distribution of APP with Aβ accumulations and plaques has been described in swollen axons associated with DAI within days of an experimental TBI [91]. This, likewise, has been reported in human TBI [87], with Aβ plaques evident within the gray matter and notably also in the white matter in close proximity to swollen axons. Together, these observations indicate that damaged axons provide a prime source of Aβ following a TBI. Such TBI-induced actions, whether occurring early or later in life, can readily increase the vulnerability of the brain to other neurodegenerative events as detailed in the ‘two hit’ hypothesis of the ‘Latent Early-life Associated Regulation’ (LEARn) model of Lahiri et al., [92, 93], whereby genetic and environmental risk factors combine in an epigenetic pathway to trigger the etiology of a later neurobiological disorder (such as TBI leading to AD). Interestingly, the direct infusion of an anti-APP antibody into the affected brain region following TBI in rats resulted in a reduction in neuronal loss, less astrocyte activation, a smaller region of brain damage and less behavioral impairment than was evident in vehicle treated TBI animals [94], supporting a reduction in APP as a therapeutic strategy worth investigating.
    Similarly, TBI has been reported to alter the distribution of α-Syn, and post-translationally modified it. An abnormal accumulation of α-Syn has been noted in axonal swellings and dystrophic neurites in TBI brains, with the creation of nitrated as well as conformationally modified forms. In rodent TBI models, striatal axons exhibit the most extensive accumulations of α-Syn forms [87]. Albeit the role of this synaptic protein requires greater elucidation, such TBI-induced changes are likely to impair its physiological role, and potentially induce a pathological one. Thus mechanisms to lower α-Syn may be of importance.
5. (−)-Phenserine augments neurogenesis:
  Extensive studies have demonstrated that neurogenesis continues to occur throughout life within key areas of the brain that include the subventricular zone (SVZ) of the lateral ventricles and the subgranular zone (SGZ) of the hippocampal dentate gyrus (DG) in rodents, in non-human primates as well as in humans [95–97]. Newly generated neural stem cells (NSCs) can differentiate into functional mature neurons and integrate into neuronal networks, including those involved in cognitive function [98, 99]. Ischemic brain injury as well as TBI stimulates the proliferation of NSCs localized to the SVZ and SGZ of adult brain, and the resultant newborn cells can migrate to damaged brain areas to potentially differentiate into mature neuronal cells [100, 101]. However, the process of neurogenesis is not particularly efficient, and is impaired by numerous factors initiated and amplified by ischemia and TBI, such as the presence of neuroinflammation [102]. Strategies and in particular, drugs that enhance neurogenesis therefore hold the potential to mitigate TBI and other neurodegenerative disorders.
    First, Fig. 9 demonstrates, (−)-Phen enhances neural precursor cell viability in cell culture – increasing neurosphere size and augmenting their survival. Second, in cellular and animal studies high levels of APP (which are elevated by TBI as well as in AD) induce the differentiation of NSCs towards a glial phenotype, and away from a neuronal one. This action is reversed by (−)-Phen [103]. Third, (−)-Phen elevates neurotrophic factor levels in brain – as assessed by measuring BDNF, a key regulator of neurogenesis [78]. In both wild type and AD transgenic mice; administration of (−)-Phen analogs has been demonstrated to augment neurogenesis [78, 104] and, notably, enhance the survival of neurospheres as well as neuronal cells in culture [78].
6. Protection of neurons from anoxia:
  The most consistent postmortem discovery in fatal head injury is the presence of cerebral ischemia [105, 106], which appears to be a key outcome predictor. Whereas there are numerous studies documenting reductions in cerebral blood flow in models of severe TBI where there is significant tissue and microvascular failure consequent to endothelial swelling, perivascular edema, and microthrombosis, particularly contiguous to focal lesions, its impact on mild and moderate TBI has remained more difficult to conclusively define. However, it is becoming increasingly appreciated that tissue hypoxia following a TBI occurs in a widespread manner in the brain, including within regions that appear to be structurally normal. Additionally, cerebral tissue hypoxia seems to arise independent of ischemia, sometimes in areas of no overlap, which suggests a microvascular etiology. The measurement of brain tissue PO_2_, particularly in humans, by using oxygen 15-labeled positron emission tomography (^15^O PET) has recently provided definitive evidence of cerebral ischemia occurrence after early TBI [107], which may persist for up to a week after injury. Hence diffusion hypoxia in seemingly normal tissue, distinct from macrovascular ischemia in injured tissue, provides potential targets in TBI for neuroprotective strategies.

**Fig. 9:**
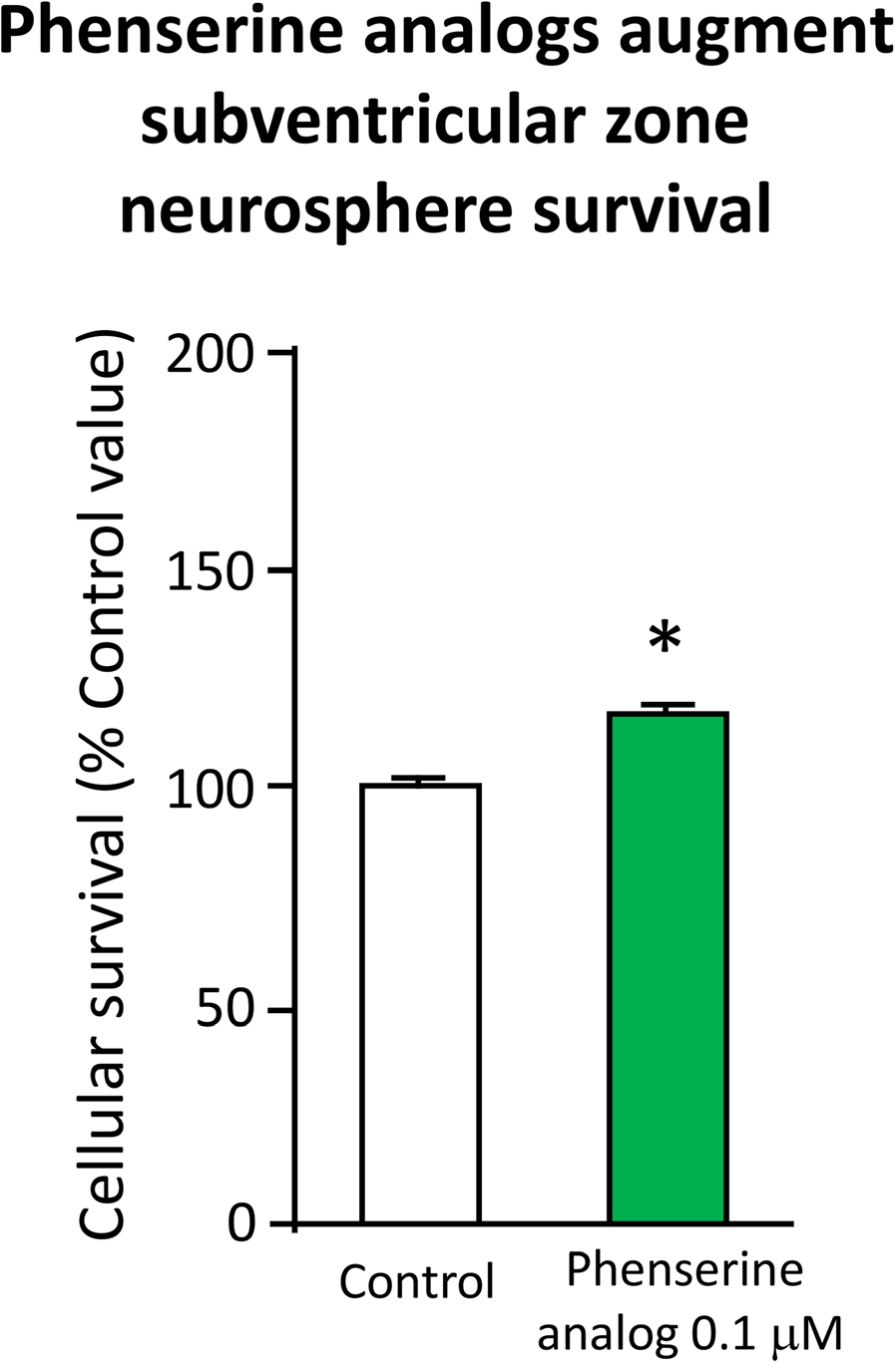
Primary SVZ progenitors cells were isolated from the lateral and medial ganglionic eminence of mouse embryos at embryonic day E13.5, and following trituration to a single cell suspension were grown as neurospheres for day 6 or 7 in vitro in the presence and absence of (−)-Phen analogs (0.01 μM) – which increased cell survival [78, 104]
    Evaluation of (−)-Phen in a classical rodent model of ischemic stroke has been undertaken by two separate research groups to appraise its protective actions in conditions of anoxia. In anesthetized male Sprague-Dawley rats with their right middle cerebral artery ligated and the common carotids clamped to induce focal infarction in the cerebral cortex after 60 min of ischemia, treatment with (−)-Phen (1 mg/kg/day) for four days, compared to placebo, reduced the area of infarction as assessed by digital scanner evaluation of brain slices (*p* = 0.001). This neuroprotective activity, in which anoxia leads to a focal lesion is supportive of beneficial actions in mild to moderate TBI, in which anoxia is considered less severe than in middle cerebral artery occlusion-induced experimental stroke.
7. Counters Cholinergic losses from Nucleus Basalis of Meynert (NBM) Injuries:
  One important pathological loss following head injury results from trauma to the basal midbrain and loss of cholinergic cells located in the NBM and/ or loss of axons providing cholinergic input to the cerebral hemispheres, hippocampus, and other critical brain structures. (−)-Phen has demonstrated efficacious benefits in the presence of NBM cell losses, an early feature of AD neuropathology that leads to elevated levels of APP and Aβ [82, 108].
    Illustrated in Fig. 10 is the AChE inhibition induced by (−)- Phen and its metabolites achieved after a single acute administration of (−)-Phen to rodents [109]. As discussed above, (−)-Phen is a highly potent inhibitor of AChE (IC_50_ = 22-36 nM) in plasma and brain, as are its N1-nor and N1,N8-bisnor metabolites [74]. In rats, at a dose of 1 mg/kg, (−)-Phen achieved a maximal inhibition of 73.5% at 5 min, and this only gradually decreased to 43% at termination of the study at 8 h, with an apparent t_1/2_ = 8.25 h (Fig. 10).

**Fig. 10:**
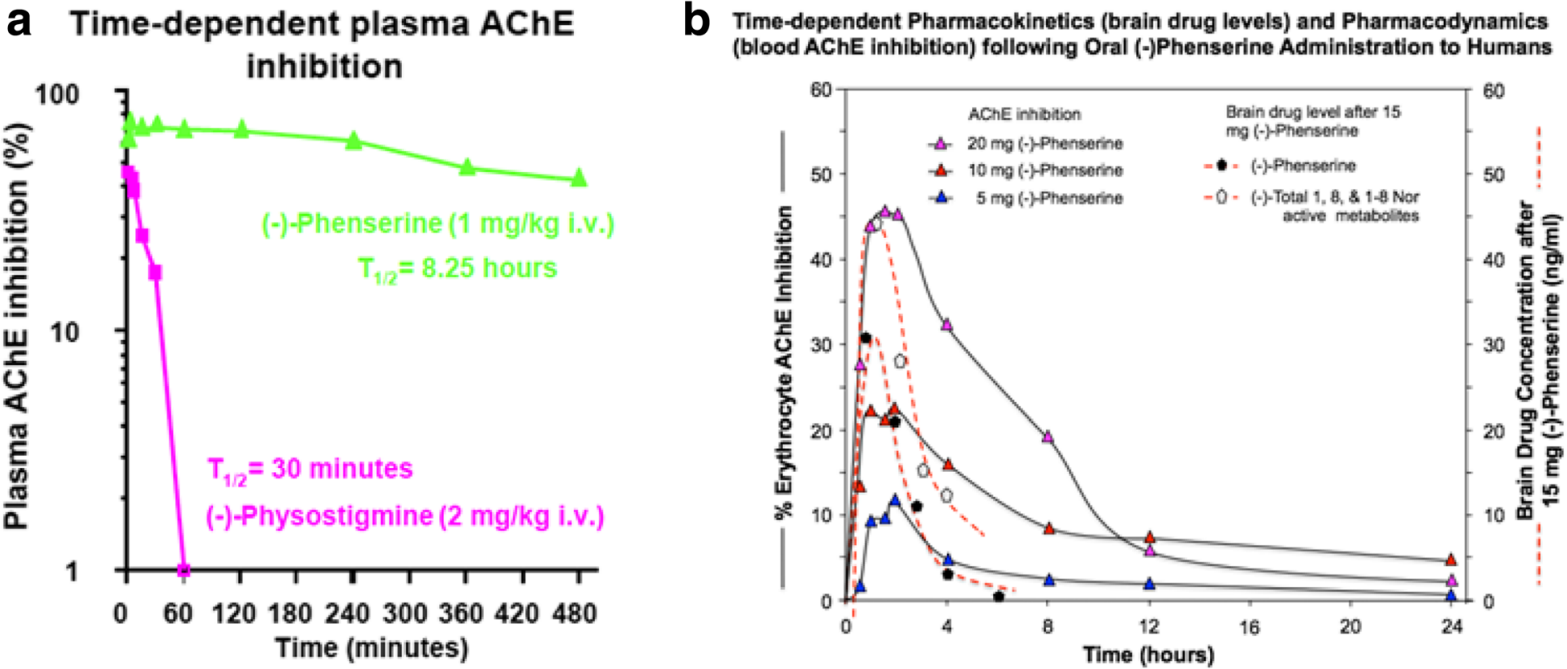
**a** and **b**. A time-dependent plasma AChE inhibition achieved by (−)-Phen in anesthetized rats following a single dose, in which cholinesterase inhibition was achieved by the combined action of (−)-Phen and its primary metabolites. (−)-Phen and active metabolites readily enter the brain (see Fig. 3), and thereby induce brain AChE inhibition and elevate acetylcholine levels [110]. By contrast, (−)-Physostigmine at a higher dose achieves lower plasma AChE inhibition, has less brain uptake than (−)-Phen, is short-lived in vivo, and is associated with greater adverses actions [72]. **b**: Time-dependent plasma AChE inhibition and predicted brain pharmacokinetics of (−)-Phen and primary metabolites in humans after single acute dosing
    Notably, these levels of AChE inhibition reflect the drug plus metabolite concentrations and result in elevated brain levels of ACh [109]. As noted in Fig. 3, (−)-Phen after oral dosing is first-pass metabolized by hepatocytes into three active (−) compounds: the N-1 Nor-, N-8 Nor-, and N-1,N-8-Bisnor-phenserine derivatives, which can all readily enter brain. Thus any given initial concentration of (−)-Phen will provide longer AChE inhibition effects than other non-cholinergic mediated drug and metabolite effects. In Fig. 10b, (−)-Phen inhibits AChE in a dose-response relationship in humans [72] that has been shown efficacious in improving cognition lost in AD where NBM lesions cause ACh deficiencies and at least partially reversible cognitive losses [73].
8. Preservation of visual memory in mTBI mice [58]:
  In Fig. 11, as evaluated by the novel object recognition (NOR) test at 7 days following a mild concussive TBI (a 30 g free falling weight from 80 cm striking a 30 g mouse on the left side of the head in the area of the parietal cerebral cortex above the hippocampus), (−)-Phen at two clinically relevant doses (2.5 and 5.0 mg/kg BID for 5 days initiated after mTBI) mitigated mTBI-induced cognitive impairment [58]. These TBI conditions (a 30 g weight and 30 g mouse) were created to mirror a human falling on their head from a three foot fall, and are considered mild concussive injuries [111], which certainly instigate neuroinflammation [112]. Notably, the rapid metabolic degradation of (−)-Phen and metabolites ensures that no pharmacological concentrations are evident in brain during cognitive evaluations performed 2 days and later after cessation of (−)-Phen dosing, thereby ensuring that mitigation of TBI cognitive deficits are not symptomatically induced by (−)-Phen’s known cholinergic actions.
9. Preservation of spatial memory in mTBI mice [58]:
  In Fig. 12, Y-maze testing was used to evaluate spatial memory and performed 7 days and after head injury. The mitigation of mTBI-induced deficits by (−)-Phen in the Y-maze cross validates the beneficial actions of the agent in the NOR paradigm, described above.

**Fig. 11:**
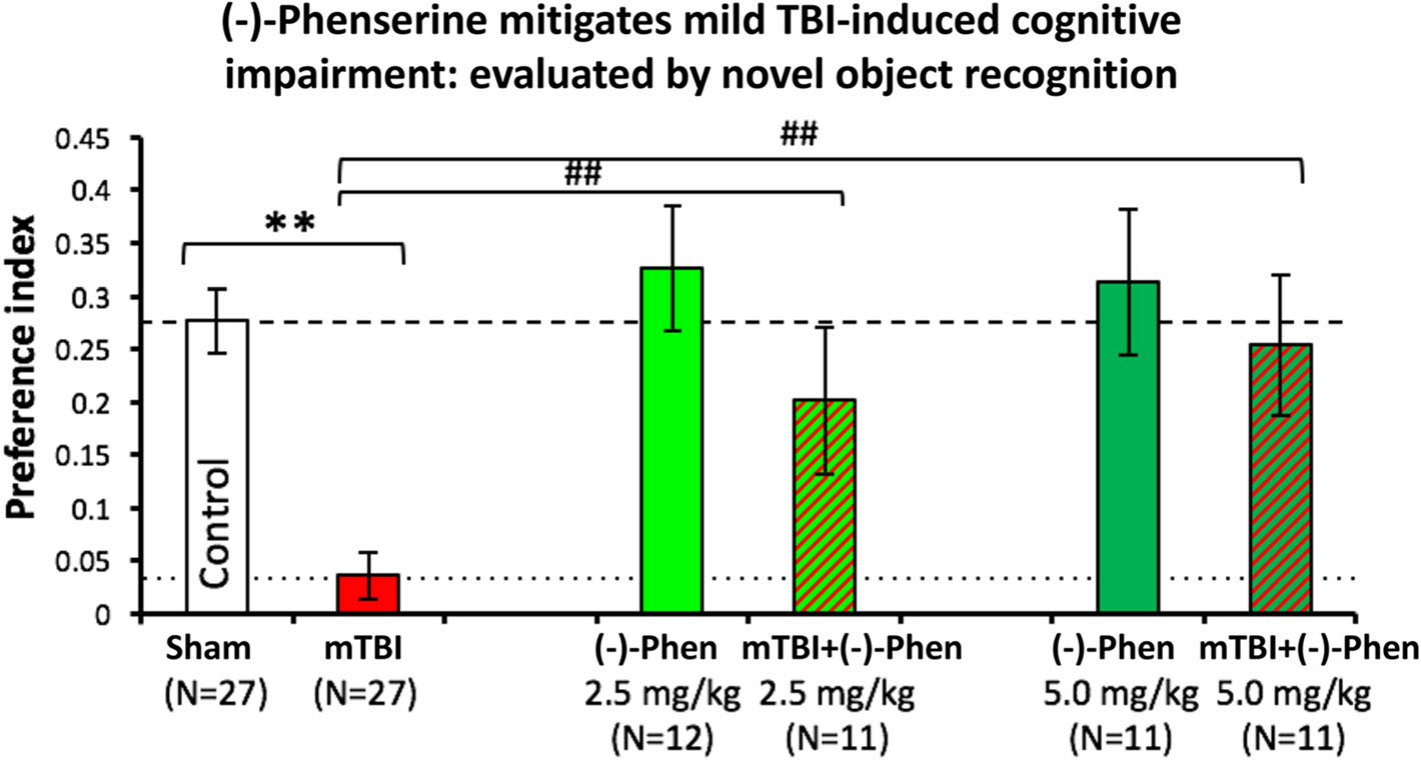
mild TBI mice demonstrate a deficit in visual memory compared with control uninjured (Sham) animals (**p < 0.01), in which (−)-Phen administration significantly ameliorated (at both doses **p < 0.01 vs. mTBI alone) [58]. A washout period of 2 days before cognitive evaluation ensured no confound in relation to any direct action to improve cognition. These data are thus interpreted as evidence for an effect of Phen against post injury pathology allowing reduced cognitive deficits in (−)-Phen treated animals (mTBI: mild TBI, Phen: (−)-Phen)
10.  Antioxidant action by augmenting endogenous antioxidant proteins [58]:
  Our prior studies highlighted in Figures illustrated above, additionally demonstrated in brain subjected to mild TBI and treated with (−)-Phen that markers of oxidative stress Thiobarbituric Acid Reactive Substances (TBARS) were reduced vs. mTBI alone, when evaluated in hippocampus 5 and 14 days after injury. This reduction in oxidative stress was consequent to a (−)-Phen induced upregulation in the activity/expression of the endogenous antioxidant proteins superoxide dismutase [SOD] 1 and 2, and glutathione peroxidase [GPx] [58].

**Fig. 12:**
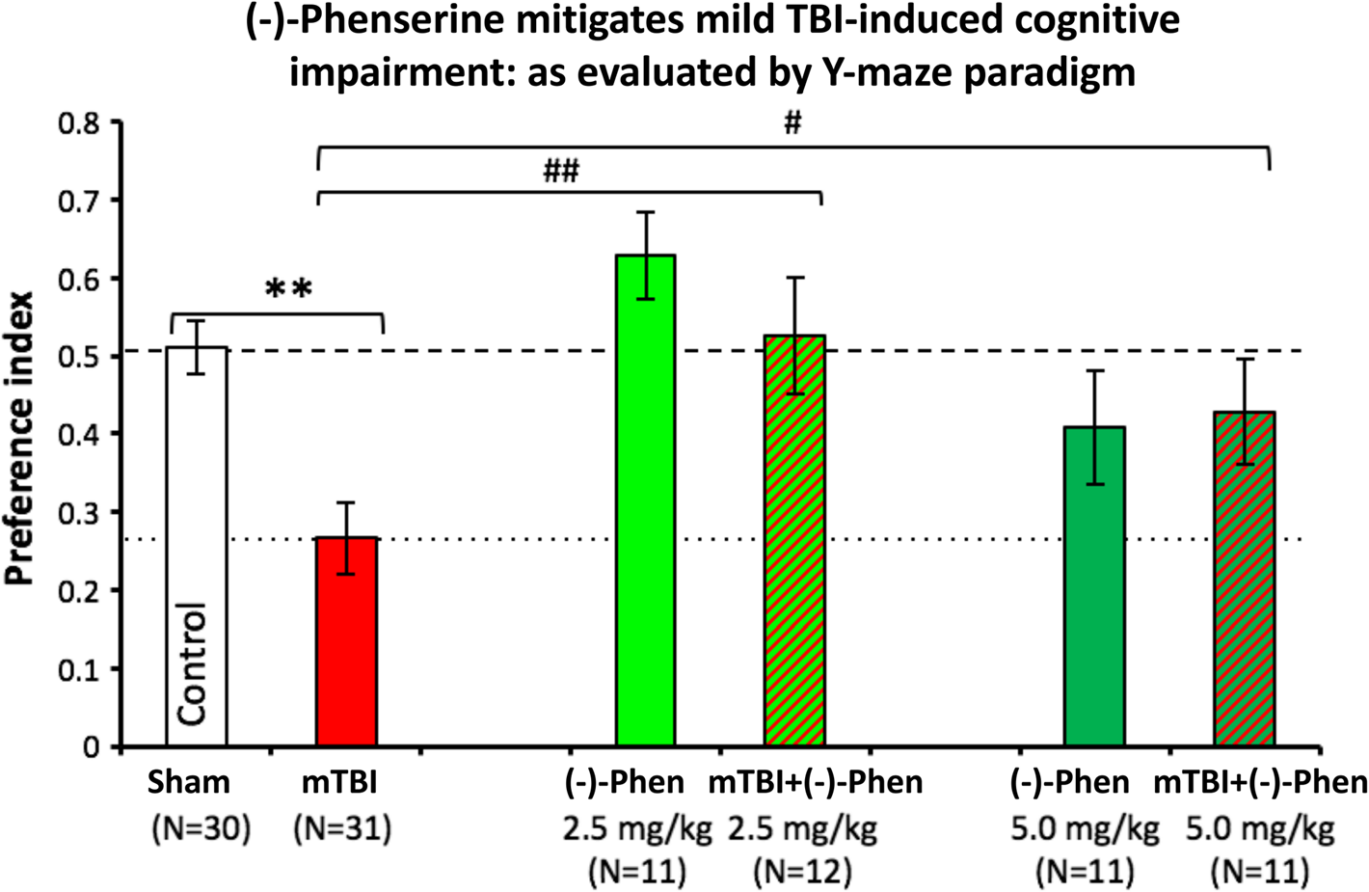
Y-Maze was assessed two days after (−)-Phen washout, evaluating two clinically translatable doses (2.5 and 5.0 mg/kg BID × 5 days) initiated post mTBI. Whereas mTBI challenged mice demonstrate a significant deficit in spatial memory vs. control uninjured (Sham) animals (**p < 0.01). (−)-Phen administration significantly ameliorated this deficit (##*p* < 0.01 for 2.5 mg/kg and #*p* < 0.05 for 5 mg/kg vs. mTBI alone) [58]. Likewise, these data are interpreted as evidence for a positive effect against post injury pathology allowing reduced cognitive deficits in (−)-Phen treated animals (mTBI: mild TBI, Phen: (−)-Phen)
    These studies demonstrate that clinically relevant doses of (−)-Phen can provide a unique and broad range of beneficial pharmacological actions that may favorably impact the programmed cell death that results following a TBI, with such apoptosis being a common feature across many neurodegenerative disorders. (−)-Phen illustrates how a drug that was opportunistically developed to supplement cholinergic activity in AD, and that has proven well-tolerated and provided a consistent evidence of efficacy [73, 113], can – consequent to its more recently discovered important broad spectrum of pharmacological actions – be optimized to not only provide potential efficacy in TBI, but also provide a pharmacological tool to understand how TBI can lead to AD. It has become increasingly clear that multiple processes combine together following an insult (whether an acute TBI or a chronic degenerative disorder such as in AD or PD) to induce the programed cell death of neurons. The modulation of (i) inflammation either directly or via cholinergic mechanisms, (ii) oxidative stress, (iii) neurosphere/NPC apoptosis/survival, (iv) glutamate excitotoxity, and (v) APP/Aβ/α-syn over-expression, as well as ability to augment endogenous trophic factors like BDNF and stimulate other such mechanisms, provides a means to both limit cell death and optimize endogenous regenerative actions. Clinical trials in TBI and AD of experimental drugs that act via a single mechanism only, such as anti-inflammatory or Aβ lowering approaches, have failed to address the full range of pathologies that lead to neuronal loss and cognitive impairment. (−)-Phen’s described activation of multiple pathways, including the augmentation of endogenous antioxidant, neurotrophic, neuroprotective, anti-inflammatory, pro-angiogenesis, APP/Aβ/α-syn-lowering as well as cholinergic and others provide neuroprotection across multiple animal models. The revelation of these multiple activities of (−)-Phen and analogs over many years exemplifies how initial notions of a drug’s mechanism of action may mislead investigators away from its full spectrum of benefits for human health.

## Conclusion

This overview provides a broad horizon of mechanisms linked to animal models and human data supportive of drug interventions having potential clinical efficacy against TBI. Many problems hinder progress identifying the mechanisms behind the interesting potential of these and other drugs and their efficacy. The criteria for identifying that a concussion has occurred does not necessarily capture head injuries with even more minor symptomatology, which may be associated with later unfavorable consequences. The duration of impairments from concussions prove highly variable and only some affected persons go on to display a post concussive syndrome, later neurological impairments, or the serious complication of chronic traumatic encephalopathy. In spite of these and other difficulties, the availability of diverse animal models with face validity for human concussions/TBI, the many affected patients, and the responsiveness of animal models and humans to the drugs we have reviewed give medical research a chance to help resolve the conundrum of TBI decisively and hopefully better define the pathologies most closely associated with the neuronal dysfunction and deaths behind post-concussive/ TBI injuries. Perhaps it is time to develop new peripheral, blood accessible, markers of TBI pathologies so that investigators can recruit human subjects for studies of TBI mechanisms. In that way we may answer why many species, used as animal models, benefit from candidate treatments for concussions while these drugs fail to meet regulatory requirements for registration for use in humans.

## Abbreviations

(−)-Phen: (−)−Phenserine
MWM: Morris Water maze
NAC: N-acetyl cysteine
TBARS: Thiobarbituric Acid Reactive Substances
TBI: Traumatic brain injury
